# The vulnerabilities of lives: Zika, women and children in Alagoas State, Brazil

**DOI:** 10.1590/0102-311X00032020

**Published:** 2021-01-11

**Authors:** Ilana G. Ambrogi, Luciana Brito, Debora Diniz

**Affiliations:** 1 Programa de Pós-graduação em Bioética, Ética Aplicada e Saúde Coletiva, Fundação Oswaldo Cruz, Brasília, Brasil; 2 Anis – Instituto de Bioética Direitos Humanos e Gênero, Brasília, Brasil; 3 International Planned Parenthood Federation, London, U.K.; 4 Universidade de Brasília, Brasília, Brasil

**Keywords:** Zika Virus, Human Rights, Social Inequity, Women’s Rights, Vulnerable Populations, Zika Virus, Direitos Humanos, Iniquidade Social, Direitos da Mulher, Populações Vulneráveis, Virus Zika, Derechos Humanos, Inequidad Social, Derechos de la Mujer, Poblaciones Vulnerables

## Abstract

Until 2015, Zika was mostly unknown in Brazil and in the world. Since then, the Zika virus has been found to be vertically transmitted and to cause congenital Zika syndrome (CZS). This study aims to describe and analyze the vulnerabilities of the women and children most affected by the Zika epidemic in Brazil. Alagoas has the lowest Human Development Index in Brazil and one of the highest rates of adolescent pregnancy. Between December 2016 and March 2017, interviews were conducted with 54 women with children affected by Zika. The interviews had two components: a narrative-oriented conversation and a semi-structured questionnaire. This comprehensive mixed methods case study represented 45% of the confirmed CZS cases and 20% of the cases under investigation in the state at that time. The women are predominantly Afro-Brazilian; most experienced their first pregnancy during adolescence, and had little education. Many were not covered by social protection programs and were not receiving adequate health care. The rights and needs of these women and children are impacted by a systemic lack of access to services and medications. There is inadequate transportation to services that many families depend on. Discrimination against their children with disabilities is a new and complex concept in their lives. The Zika epidemic has compounded rights violations in their lives and worsened their social and economic layers of vulnerability. There is an urgent need for a robust public response to guarantee the rights of these women and children and to implement mechanisms to prevent and eliminate their vulnerabilities.

## Resumo

ResumenAté 2015, o Zika vírus era praticamente desconhecido no Brasil e no mundo. Desde então, descobriu-se que o vírus é transmitido verticalmente, da gestante para o feto, e que causa a síndrome congênita do Zika vírus (SCZV). O estudo tem como objetivo descrever e analisar as vulnerabilidades das mulheres e crianças mais afetadas pela epidemia do Zika no Brasil. Alagoas é o estado do Brasil com o Índice de Desenvolvimento Humano mais baixo, e com uma das maiores taxas de gravidez na adolescência. Entre dezembro de 2016 e março de 2017, foram realizadas entrevistas com 54 mulheres com crianças afetadas pelo Zika. As entrevistas tinham dois componentes: uma conversa orientada por narrativa e um questionário semiestruturado. Este estudo de casos com métodos mistos representa 45% dos casos confirmados de SCZV e 20% dos casos investigados no estado na época. As mulheres eram predominantemente afro-brasileiras, a maioria primigesta durante a adolescência e com baixa escolaridade. Muitas não estavam inscritas em programas de proteção social e não recebiam cuidados de saúde adequados. Os direitos e necessidades dessas mulheres e crianças são impactados pela falta sistemática de acesso a serviços e medicamentos. Há deficiências no transporte público até os serviços de saúde, dos quais dependem muitas famílias. A discriminação contra os filhos com a síndrome é um conceito novo e complexo na vida dessas mulheres. A epidemia do Zika agravou as violações de seus direitos e os fatores sociais e econômicos de sua vulnerabilidade. Há uma necessidade urgente de uma resposta pública robusta para garantir os direitos dessas mulheres e crianças e para implementar mecanismos para prevenir e eliminar as vulnerabilidades.

## Resumen

Hasta el 2015, Zika era desconocido para la mayoría, tanto en Brasil, como en el resto del mundo. Desde entonces, se ha descubierto que el virus del Zika se trasmite verticalmente y causa el síndrome congénito por el virus Zika (SCZ). El objetivo de este estudio es describiry analizar las vulnerabilidades de las mujeres y niños más afectados por la epidemia de Zika en Brasil. Alagoas cuenta con el índice más bajo de desarrollo humano y una de las más altas tasas de embarazo adolescente. Entre diciembre de 2016 y marzo de 2017, se realizaron entrevistas a 54 mujeres con niños afectados por el Zika. Las entrevistas estuvieron compuestas por: una conversación orientada al relato y un cuestionario semiestructurado. En aquel momento este estudio de casos con métodos combinados integrales representó un 45% de los casos confirmados de SCZ, y un 20% de los casos en investigación, dentro del estado. Las mujeres eran predominantemente afrobrasileñas, la mayoría vivieron su primer embarazo durante la adolescencia, y contaban con poca formación educativa. Muchas no estaban inscritas en los programas de protección social y no estaban recibiendo cuidados de salud adecuados. Los derechos y necesidades de estas mujeres y niños estaban afectados por una carencia sistémica de acceso a serviciosy medicamentos. Existe un transporte inadecuado a los servicios de los que dependen muchas familias. La discriminación contra sus hijos con discapacidades es una nueva y compleja vicisitud en sus vidas. La epidemia de Zika ha agravado las violaciones de derechos en sus vidas y empeorado varios de sus factores de vulnerabilidad social y económica. Existe una necesidad urgente de una respuesta pública rotunda, con el fin de garantizar los derechos de esas mujeres y niños, así como implementar mecanismos, con el fin de prevenir y eliminar sus vulnerabilidades.

## Introduction

In February 2016, the World Health Organization (WHO) declared a global emergency situation due to the effects of Zika virus infection during pregnancy. In Brazil, the Northeast Region was the epicenter of the country’s epidemic. Given Zika’s effects on embryonic, fetal, and postnatal development, the virus became a concern for women of reproductive age and particularly for those who were pregnant at the time ^1,2^. During this epidemic, the northeastern states of Bahia and Pernambuco were considered to have the highest incidence of cases ^3^. Alagoas, a small state nested between Bahia and Pernambuco and with the lowest HDI (Human Development Index) in the country ^4^, was considered a “paradox” by the Ministry of Health authorities given its low number of reported cases during the epidemic ^5^. Alagoas has similar climatic, geographical, social and economic characteristics to the heavily affected states. The claim of an unexpectedly low number of reported cases of Zika in Alagoas should raise many questions.

Currently, Brazil continues to be the global epicenter of Zika illness. There are still cases of congenital Zika syndrome (CZS) and of Zika viral illness being reported. From the onset of monitoring of the epidemic in November 2015 through December 2019, there were more than 280,000 reported cases of people with suspected Zika illness ^6,7,8^ and 18,578 reported cases of newborns suspected of having CZS ^9^. Of these newborns, 3,496 were subsequently confirmed as having the syndrome, 763 were classified as probable syndrome cases, 2,665 still remained under investigation and 638 were considered inconclusive ^9^. Nationally, according to the latest epidemiological report that reported on this, only 35% of the children with CZS are receiving early stimulation services ^10^ and 39% are not getting any specialized care ^11^. Moreover, 37% of children with the diagnosis are not even receiving routine pediatric/primary care ^11^. The majority of these children – 2,189 of them – are concentrated in the Northeast region, which is also the region that has the highest number of reported fetal, neonatal and infant deaths suspected to be related to Zika infection ^9,11^.

By December 2016, at the time this field research was conducted, the Ministry of Health announced that Alagoas had 371 reported cases of newborns suspected of having CZS, which represented 3.6% of the national total for that year. Of the total reported cases in Alagoas, 86 were confirmed to be CZS, 51 cases were still under investigation and 234 cases were ultimately discarded ^12^. Calculations using the epidemiological data available at the end of 2016 showed that Alagoas had twice as many discarded cases of newborns suspected for CZS per 10,000 live births as its neighboring state, Bahia. Alagoas had 22/10,000 live births as discarded cases while Bahia had half of that: 11/10,000. From the start of the epidemic in 2015 through the end in December 2016, 76% of all the reported suspected cases in Alagoas were either discarded or were still awaiting a final diagnosis ^5^.

The clinical criteria used for epidemiological surveillance during the public health alert were newborns with signs of microcephaly as determined by head circumference measurements. These criteria not only changed during the epidemic, due to attempts to establish an international standardization, but they were also hard to implement ^13,14^. In addition to the complexity of the situation, federal and state governments developed different response policies for identifying suspected cases ^15,16^. The expectation that fetal alterations would be identified by prenatal ultrasound also does not account for the realities of regional and local public health services. Public health services only guarantee one ultrasound during pregnancy, which is usually done in the early stages of pregnancy, a period when the effects of Zika are not usually detectable ^17,18,19^.

In its first protocol for epidemiological surveillance of microcephaly due to Zika virus infection published by the Alagoas State Department of Health, a non-contrast computerized tomography (CT) of the head was required for newborns with microcephaly suspected to be due to Zika. This differed from the national protocol, where a transfontale ultrasound not only was an alternative to the head CT but was also considered to be the preferred method, given the exposure to radiation in the CT exam and the frequent need for sedation. Although Alagoas has since updated its protocol to include transfontale ultrasound as a possible imaging option, the protocol still states that head CT is the preferred method, since it is more readily available in the state than ultrasound ^15,16^. Alagoas has only two public hospitals with tomography machines and the wait time for an exam is several months ^20^.

All of these obstacles to access care and to obtain a diagnosis and treatment have a significant compound effect on the preexisting vulnerabilities of the most affected population ^21^. In this study, vulnerability is understood according to a practical sphere; it is relational, dynamic and context-dependent. Vulnerabilities can be multiple and distinct. They can be layered and potentiate further vulnerabilities and human rights violations. Each preexisting vulnerability can increase the chances for another, and this can have a cascade and cumulative effect ^21^. The analysis of layers of vulnerability must include the social context and, in the case of women and children affected by Zika, it should consider previously identified aspects of their lives. Social, economic and gender inequalities place people in situations that enhance the layers of vulnerabilities, particularly when living in places that have been historically ruled by oppressive patriarchal systems and that are underdeveloped.

In order to minimize and/or eradicate vulnerabilities, it is important to study and determine which situations can trigger the cascade layers of vulnerability ^21^. The life circumstances of those most affected by Zika remain unchanged, there continues to be new cases of CZS reported ^8^ and the recent political change in Brazil has significantly impacted the chances of this population obtaining health care and other resources necessary for their well-being. This study aims to describe and analyze the layers of vulnerabilities of the women and children most affected by the Zika epidemic in Alagoas. It shows how the aftermath of the Zika epidemic not only intensified many of their social and economic vulnerabilities but also how the outbreak triggered a cascade effect that reaffirms the potential of epidemics to become “poverty traps” for those affected ^22^.

## Methods

This was a comprehensive mixed methods case study conducted in the Brazilian state of Alagoas. This study is part of a larger research project and some of its findings were discussed in the report *Zika in Brazil – Women and Children at the Center of the Epidemic* ^5^. During December 2016, our research team traveled over 800 kilometers and visited 21 municipalities in the state. Data collection was finalized in March 2017. Active search for families occurred via different sources: official epidemiological surveillance records (municipal, state, and federal), WhatsApp (a popular mobile communication application) groups of mothers and caregivers of children with CZS, health teams at reference centers, and community contacts. Official state records did not provide names for the women affected, only the names of the municipalities where the cases were recorded. Using this information, the research team traveled to the municipalities – most of which are remote and sparsely populated. Public transport was also scarce, and motorcycles were commonly used as taxi transportation. Upon arriving in the municipalities, the researchers asked drivers at these motorcycle taxi stops whether they knew of any affected children.

The data in this article reflect the biomedical literature available at the time of the unfolding epidemic. There have since been several changes in parameters and guidelines as more information became known ^13,15^. Interviews were conducted with 54 women with children who had confirmed or suspected CZS according to the criteria at the time of their birth or during pediatric care. The interviews were individual, most at the women’s homes. Four women were interviewed on the day of their children’s consultation at the Dr. Helvio Auto University Hospital – the state’s reference center for treatment of tropical diseases.

The interviews had two methodological components. The first was a narrative-oriented conversation, with a topic guide that explored demographic elements, access to social benefits (income transfer, medications and transportation), as well as infant care and experiences of discrimination. The second methodological component was a semi-structured questionnaire that repeated some questions from the narrative-oriented conversation with emphasis on demographic elements (age, ethnicity, income, number of children, education), urbanization and housing (sewage, garbage collection, type of housing), and access to health services and early stimulation for the child. Each interview lasted approximately 60 minutes. One third of the women were interviewed more than once for adequate data collection. After transcribing the interviews, some answers were clarified or confirmed over the phone. Data analysis was based on thematic analysis, enabling the preparation of a conceptual description ^23^. Data were coded and grouped by concepts for the formulation of themes of what emerged from the data, without being previously conceived ^24^.

Of the 54 female participants, 39 had a child with confirmed CZS, 10 had a child who was still under investigation for Zika effects and other congenital infections, and 5 were excluded from the analyses due to notification errors. This sample represents 45% of the confirmed cases of CZS and 20% of the cases under investigation in Alagoas at the end of December 2016. Of the total number of municipalities with confirmed cases (40), this study covered 52% (n = 21). After initially disaggregating the data of the 2 groups of women by age, ethnicity, level of education, access to social benefits, there were no significant differences between the 2 groups of women interviewed. Consequently, the data on women with children with a confirmed diagnosis and those with children whose cases were still under investigation were combined for the analysis presented here.

The exclusion criteria were as follows: if the newborn had diagnostic imaging with a normal result; if full-term newborn head circumference was 33cm at birth (the cutoff for microcephaly in 2016 was 32cm, lowered from 33cm in 2015) ^13^; if the women did not have any record or recollection of Zika illness during pregnancy; if the child had not shown any symptoms or signs of developmental delay or neurological disorder according to pediatric records; and if there was diagnostic confirmation by more than one medical professional that this was not a case of CZS.

Ten children without definitive diagnosis were included in the analysis because they fell into at least one of the following 3 inclusion categories: they did not meet the Brazilian Ministry of Health criteria to be considered a discarded case; they were classified as having a congenital syndrome other than Zika but imaging was suggestive of congenital infection that did not match the suspected diagnosis; children born in Alagoas in late 2015 or during 2016 who presented with multiple signs and symptoms that required similar if not identical living and care needs as children with CZS.

Additional data on reported cases of CZS in Alagoas were collected from the Brazilian Ministry of Health and from the Brazilian Ministry of Social and Agrarian Development. This data collection complied with the Law of Access to Information (LAI) – *Law n. 12,527/2011*, which regulates the right to obtain access to public information. Available data on all cases reported in the state between 2015 and April 2017 were requested. These data contained disaggregated information on the women’s ethnicity, age, place of residence, and social assistance for their children. These data were cross-referenced with the data we collected in the interviews and analyzed as presented in the results section.

This study was funded by the Wellcome Trust and DFID, 206021/Z/16/Z. Prior to the start of any field activities, the research protocol was reviewed and approved by the University of Brasilia Research Ethics committee for humanities and social science – CAAE: 63604016.4.0000.5540. This study complied with the International Ethical Guidelines for Health-related Research Involving Humans. Oral Informed consent was preferred given the low levels of literacy among the study participants and the low risk level of the research intervention. In order to ensure immediate benefit sharing, at the end of each interview the families were informed about the social benefits available for their specific needs. The team also answered participants’ questions related to access to child health, sexual and reproductive care, or social welfare assistance and provided information regarding available services when appropriate. Considering the importance of benefit sharing at the time of the research intervention, this ethical approach was enabled by the presence of a social worker, a lawyer, a physician, a nurse and a community health agent in the research team.

## Results and discussion

### Participants’ profile

The participants’ age ranged from 14 to 43 years. However, only 3 women were past their twenties. About half of the women, 51% (n = 25), became pregnant and were affected by the epidemic during their adolescence. Adolescent mothers were aged 14 to 19 years. Six of the women became pregnant before their 15th birthday. Taking into account previous children, 75% (n = 37) of the women interviewed had first become pregnant during adolescence. According to data from the Brazilian National Information System on Live Births (SINASC), this number is about three times higher than the state’s adolescent pregnancy rate (26%), which is already among the highest in the country, and around four times the national rate (18%) (http://tabnet.datasus.gov.br/cgi/deftohtm.exe?sinasc/cnv/nval.def, accessed on 18/Feb/2020).

In addition to being very young, most women were Afro-Brazilian (80%, n = 39), which is higher than the state’s racial distribution of Afro-Brazilian persons (72%) and much higher than the national ratio (53%) ^25^. As for the level of formal education, young age seemed to compound these women’s vulnerabilities. Three of the women (6%) were illiterate, which is higher than the national incidence of illiteracy and thus suggests the level of precariousness of their lives. According to the *Brazilian National Household Sample Survey* (PNAD), the national illiteracy rate is 1.4% for women in a comparable age range – 24 to 29 years ^25^. Again, lower than expected levels of education. For almost half of the adolescents, the level of formal education was very low: 52% (n = 13) had not completed elementary school. The national rate of adolescents aged 17 to 19 years with incomplete elementary education is less than 15% ^25^. Generally, their education is disrupted because of poverty and/or early pregnancy and the failure of social welfare policies.

### Income and lack of access to social welfare programs

The analysis of income information took into consideration that there is often resistance in disclosing incomes due to both fear and embarrassment. The fear is that the information may be possibly misused or misrepresented, thereby risking the loss of social benefits, which are allotted based on income. Questions related to income in contexts of extreme poverty can also provoke shame and timidness. However, despite these limitations, the different sources of data confirmed that the participants were living in extremely vulnerable socio-economic conditions. Yet, despite their poverty, several families did not receive the Bolsa Família benefit, one of the main social welfare programs for poor families in Brazil.

There are two major social welfare programs in place for these families: Braziliam Income Transfer Program (*Bolsa Família*) and the Continuous Cash Benefit (*Benefício de Prestação Continuada* – BPC). Braziliam Income Transfer Program is an income transfer program for very poor families with children younger than 17 years at home ^26^. BPC is a Brazilian income transfer benefit equivalent to a minimum wage paid monthly to every person with a disability whose family income per capita is less than ¼ of the monthly minimum wage salary (i.e. USD 50 per person/month) ^27^. Despite the many vulnerabilities of their lives and having children with disabilities, most families in the study did not receive the BPC (63%, n = 31). The women described significant barriers to obtaining this benefit to which they were entitled. First, the medical and social service documents that are required to register for the benefit program were considered to be excessive and cumbersome to obtain. The women also frequently mentioned the lack of transportation as an insuperable barrier to completing the required bureaucratic process.

As for the Braziliam Income Transfer Program, there were primarily two, interrelated, reasons for the low number of families registered for the program, despite most of them meeting its criteria. First, women reported that the local social security officers informed families that they were not allowed to receive the two benefits (BPC and Braziliam Income Transfer Program) for the same child/family; second, families did not want to risk losing the BPC, which provides a larger amount of money than Braziliam Income Transfer Program, by trying to also obtain the Braziliam Income Transfer Program. The reality of how these benefits are understood and in fact granted differ from the actual criteria as established by law. These benefits are not mutually exclusive and the Braziliam Income Transfer Program income amount is not supposed to be used in calculations of household per capita income when determining eligibility for BPC ^27,28^. The Braziliam Income Transfer Program is a benefit thought for the entire family which usually includes other children, while the BPC is an assistance for the unmet financial needs of a specific individual.

Of the 25 adolescent mothers with a child confirmed or under investigation for CZS, 40% (n = 10) were not participating in any income transfer programs: neither BPC nor Braziliam Income Transfer Program. When the programs were analyzed separately, it was found that 76% (n = 19) of the adolescent mothers did not receive BPC for their eligible child and 44% (n = 11) did not receive Braziliam Income Transfer Program even though they were entitled to both. The highest inequity of benefits allotted were among adolescent mothers with children with CZS. Only 1 in 4 of those entitled to participate in an income transfer program was registered.

It is at the intersection between the different requirements of the different programs that the adolescent women’s vulnerabilities become more acute: out of school, they are not eligible for Bolsa Familia for their household because the criterion of inclusion for the program is school enrollment; adolescent mothers without record of civil emancipation were not understood as subject of rights for their children with CZS. The alternative for some adolescents was to register the child as a dependent of their grandparents, despite living in different homes.

More than half of the women had been engaged in paid work before their pregnancy (53%, n = 26), and 76% of them had not returned to work after childbirth. Their jobs were in agriculture, retail, education or as housemaids. All families, to different degrees, reported experiencing a decrease in their standard of living and an increase in expenses after their child’s birth, including with transportation and health care. In this sense, BPC is decisive for the survival of these families and the guarantee of care for the children. The requirement of an extremely low level of income for families to access the benefit has significant consequences on the families’ well-being and access to fundamental rights. It caused women to feel trapped and conflicted about returning to paid work.

### Need for transportation to services

In most municipalities visited, there was no public transportation to either of the two state’s reference centers for pediatric early stimulation therapy, which are located in the cities of Maceió and Arapiraca. Over half of the women (55%, n = 27) depended entirely on transportation provided by their municipality, such as ambulances or chartered cars, to take their infant to weekly 30-minute physical therapy/occupational therapy/early stimulation sessions. Of the remaining women, 45% (n = 22) reported that given the lack of transportation services provided by the municipality, they were unable to take their children to the prescribed early stimulation sessions as recommended by the health care professionals. On average, round-trip commute to services was 3 hours. The families with the shortest commute were those who lived closest to the reference centers. The more remote families traveled up to 6 hours round-trip for the 30-minute early stimulation therapy session.

Many remote municipalities of Alagoas have scarce or, in many cases, nonexistent public transportation. This study showed that half of these children depend on the local municipal government for transportation in order to get to the needed services. In addition, there were abundant reports of transportation involving safety issues and mistreatment. There is a crucial need for outreach to remote, low-income areas where many of the participants live. As mandated by Brazilian law, these families are entitled to reliable and safe transportation for themselves and/or for providers to be able to visit their homes. Home visits or mobile medical vehicles with a multidisciplinary team should be considered for families affected by Zika and others with similar, multiple vulnerabilities ^29^.

### Need for medications and supplies

Despite the Brazilian legislation recognizing health as a right of its citizens and a government’s responsibility, of the 23 children (47%) on daily prescribed medication, only 6 received medication from public health services. There was a systemic lack of medications, in particular anti-epileptic drugs, in public health services and pharmacies. About half of the families, 53% (n = 13) who were prescribed medications to control seizures reported not having the financial means to buy the medication without some sort of assistance. There have been cases of children who experienced uncontrolled seizures and had to be hospitalized for prolonged periods of time because of complications such as aspiration pneumonia. At the time of data collection, none of the women interviewed whose child had a need for visual aid had received eyeglasses via the public health system. The participants also reported recurrent issues of inadequate staffing and/or shortage of supplies in the local health care facilities. These reports echo the findings of the most recent official epidemiological report, which showed that over 35% of the children with confirmed CZS are not getting specialized care and/or even routine pediatric care ^11^.

### Discrimination and the meaning of having a “special child”

None of the women interviewed described their child as having a disability or even a disease or syndrome. Rather, the women described the child who needed extra care at home and more medical attention as a “special child”. During the interviews, the word “disability” was never used as an emic category to describe the “special child”. Disability was still a category used primarily among academic and activist movements in Brazil. It did not possess an emic significance for these women. Nor did Zika or CZS. The women have instead embraced microcephaly as the term that describes the physical condition of the child who needs specialized care.

When asked “What does he have that makes him have to go to the doctor?”, the primary answer was “He has microcephaly”. To comprehend the significance of the word “microcephaly” to these women, it is necessary to consider the layers of meanings that emerge in their accounts. The first was the normalization of the child, because microcephaly was simply intended to mean “a small head”. It was common to hear “He is normal, he only has the microcephaly problem”, which could falsely suggest a misunderstanding about the meaning of the medical diagnosis. “Normal” and microcephaly was a pair that needed to be joined to confront public curiosity about the child with the “mosquito problem”. Describing the child as normal is a way of rejecting the degrading and offensive questions that nearly all of the women had heard at some point, either when the child was born or upon their return home. Women were asked whether they were going to abandon their child, be able to love the child, or thought of the child as beautiful.

Occurrences that could be described as public expressions of discrimination were commonly described by the women as acts merely based on “curiosity”. People removing hats from babies’ heads or touching them were rarely described with a language that implicates rights violations or denounces the discrimination suffered. There is a subtle layer here of cultural language and accommodation to humiliation: not all curiosity was understood as offensive, because life experiences in the community are considered to be shared experiences. To identify the curiosity of others as a form of discrimination was not an instinctual or immediate process for many women, yet as the child grows the pairing of microcephaly with “normal” becomes increasingly more difficult. It is a complex affective and moral construct about maternity, inclusion, care and struggles against discrimination.

### Final considerations

This study describes the impacts of the Zika epidemic on the lives of women and children in Alagoas. The arrival of Zika in Brazil triggered and intensified vulnerabilities that were already part of these women’s lives, due to inequalities that have long existed in Brazil. In order to minimize the negative effects of Zika, there is an urgent need for public policies to safeguard the rights of the women and children at the center of the epidemic and those who remain at most risk ^30^. A Supreme Court case requesting the protection of rights of women and children affected by Zika filed in 2016 had its hearing scheduled, after a four-year wait, suddenly during the COVID-19 pandemic in April 2020. The court dismissed the case on procedural grounds ^31^. In a time span of four years, these children and women are now living a second public health crisis, the COVID-19 pandemic, and many of their fundamental needs remain unmet ^11^.

The social and economic vulnerabilities that put women and children at risk for Zika remain. The growing children have developed different medical, social and educational needs. Children of school age need individualized educational plans as well as transport and accessibility to public spaces ^32^. The higher number of discarded cases observed in Alagoas during the aforementioned period should lead to the consideration that there might be children still in need of further evaluation. The reasons that led the Brazilian Northeast region to be the epicenter of the Zika epidemic are not fully known ^33^ – what is known, however, is that the epidemic revealed structural fragility in public policies that should have protected the most vulnerable. Rights violations were observed in several domains of the lives of women and children participants: from lack of adequate medical care and late diagnosis of the syndrome in newborns to discrimination and harassment.

Women are the principal caregivers ^34^ and are on the frontlines of the daily struggles. Loss of income has been observed in these families and the BPC, the cash transfer benefit, is considered an important financial aid ^34,35^. In March 2020, after the Federal Senate’s reversal of a presidential veto, a law proposal from 1996 requesting the raise of the minimum per capita income to qualify for this cash transfer benefit so more families could be included was finally approved ^36^. This triggered an immediate reaction from the executive branch of the government leading to an appeal to the Supreme Court for the law to be barred. The court sided with the government and suspended the new law and the proposed changes for BPC qualification never actually went into effect ^37^. Throughout the years, it is not uncommon for individual families caring for affected children to appeal to the courts requesting cash transfer benefit, the BPC, by demonstrating that their expenditures greatly surpass their family income. However, this is usually a long, difficult and bureaucratic process that adds yet another layer to their situation of vulnerability ^38,39^.

Zika not only exposed various rights violations that have been systematically disregarded but also showed how those violations exacerbate the vulnerabilities of these women and children ^40^. Even though our data was limited by purposive sampling, Brazil continues to be the global epicenter of Zika illness. The virus and the vector are still present in the country. New cases continue to be reported ^9^. In order to prevent the worsening of their living situation, we must first determine the vulnerabilities among these women and children. This study does that. It is a first step toward minimizing, preventing and even eradicating the most harmful layers of vulnerability that put this population at risk for Zika and its negative effects. Public and social health policies must attend to the needs of women and children already affected by Zika and of those at risk. It is urgent that authorities engage in both more research and action.
